# Waste Wood Particles from Primary Wood Processing as a Filler of Insulation PUR Foams

**DOI:** 10.3390/ma14174781

**Published:** 2021-08-24

**Authors:** Radosław Mirski, Dorota Dukarska, Joanna Walkiewicz, Adam Derkowski

**Affiliations:** Department of Wood Based Materials, Faculty of Forestry and Wood Technology, Poznań University of Life Sciences, Wojska Polskiego 38/42, 60-627 Poznań, Poland; rmirski@up.poznan.pl (R.M.); adam.derkowski@up.poznan.pl (A.D.)

**Keywords:** polyurethane foams, filler, wood particles, structure, properties

## Abstract

A significant part of the work carried out so far in the field of production of biocomposite polyurethane foams (PUR) with the use of various types of lignocellulosic fillers mainly concerns rigid PUR foams with a closed-cell structure. In this work, the possibility of using waste wood particles (WP) from primary wood processing as a filler for PUR foams with open-cell structure was investigated. For this purpose, a wood particle fraction of 0.315–1.25 mm was added to the foam in concentrations of 0, 5, 10, 15 and 20%. The foaming course of the modified PUR foams (PUR-WP) was characterized on the basis of the duration of the process’ successive stages at the maximum foaming temperature. In order to explain the observed phenomena, a cellular structure was characterized using microscopic analysis such as SEM and light microscope. Computed tomography was also applied to determine the distribution of wood particles in PUR-WP materials. It was observed that the addition of WP to the open-cell PUR foam influences the kinetics of the foaming process of the PUR-WP composition and their morphology, density, compressive strength and thermal properties. The performed tests showed that the addition of WP at an the amount of 10% leads to the increase in the PUR foam’s compressive strength by 30% (parallel to foam’s growth direction) and reduce the thermal conductivity coefficient by 10%.

## 1. Introduction

In recent years, people’s environmental awareness has been increasing, which has led to the search for solutions that will allow the use of technologically processed by-products. Due to the increasing development in the wood industry, waste generation is a common problem. Two by-products of wood processing are dust and wood particles. Despite the fact that research is carried out with the use of wood dust in the context of various materials, this material is still a nuisance waste. Nowadays, the most popular composite containing wood (of any form) is a wood plastic composite (WPC) [1]. Research concerning the application of WP was also conducted in order to enhance the properties of thermoplastic starch [2]; as a component in adhesive mixtures for 3D printing [3]; in concrete as a partial replacement for sand [4]; and in the production of new polyurethane foams from liquefied wood powder [5]. Wood waste can also be applied as a potential filler for loose-fill building isolation [6].

PUR represents a wide class of polymeric materials [7,8]. Polyurethane foams account for 2/3 of the world’s production of polyurethanes, and because of their numerous applications in the form of rigid, semi-rigid and flexible foams, they are continuously highly ranked among all of available foams [9]. PUR foams are the product of the addition polymerization of polyols and polyisocyanates. Catalysts, surfactants and foaming agents are also used during the production of PUR. These foams may differ in composition, density, color and mechanical properties. There are also studies where fillers were used to lower the cost and increase mechanical properties, e.g., the modulus and strength or density [10]. Polyurethane foams, particularly flexible polyurethane foams, are commonly used in the building, automotive, furniture and packaging industries [11,12,13,14,15]. These foams have many advantages, such as a wide range of flexibility, good shock absorption and high durability in use. Moreover, they can be easily formed into various shapes, and they are characterized by a low density. However, they are not cheap. It is also worth adding that there is an opportunity to easily control and manage their parameters by changing their composition or using fillers [16].

Literature data clearly indicate that the organic or inorganic fillers can significantly improve the mechanical and thermal properties of PUR composites [17,18,19,20]. The introduction of inorganic fillers such as glass fibers or carbon fibers in polyurethane foams is a well-known process [21]. The fillers strongly affect the physical properties of foam. Not only inorganic fillers are used in foam formulations, but they can also be filled with materials of natural origin, such as plant and agricultural waste. Agricultural waste has the potential to be used in the production of PUR foams as renewable bio-based fillers [17]. Członka et al., Kerche et al., and De Avila Delucis et al. [19,22,23] claimed that the use of agricultural waste in the production of PUR materials can result in a new approach in the application path and provide a new opportunity for creating a new class of green materials. Paciorek-Sadowska et al. [17] used rapeseed cake as an addition for PUR composite production. Authors observed that 30–60% incorporation of rapeseed cake significantly influenced the apparent density and mechanical properties. Modified sunflower cake was added to PUR foam, and it was found that the addition of plant waste had an impact on the improvement of the mechanical properties, while it did not cause a significant deterioration of the insulating properties [24]. Funabashi et al. [21] researched the effect of filler types on mechanical properties of a rigid polyurethane composite. For this purpose, authors used particles from plant wastes (e.g., bamboo powder, coffee grounds, wood meal and cellulose). Zieleniewska et al. [25] obtained the composites of rigid polyurethane foams (RPURF) with the use of waste hazelnut shells, suitable for applications in the cosmetics industry. Moreover, rice plant wastes were used as a reinforcing material in the concentrations of 5%, 10%, 15% and 20% [26]. According to the literature, wood waste can also be a valuable raw material for the production of biocomposite PUR foams. According to Augaitis et al. [6], their application is beneficial due to their high availability, low cost and content of free hydroxyl groups capable of reacting with isocyanate groups. These authors showed that a biocomposite PUR foam with an apparent density of 150 kg/m^3^ and a PUR to pinewood sawdust ratio of 0.7 is characterized by very good physical and insulating properties and high strength. In turn, in this paper, an innovative solution has been proposed to use waste wood particles from primary wood processing as a filler for PUR foam. To the best of our knowledge, this type of filler has not been used in the process of producing insulating PUR foams with open-cell structure so far. It seems that such operations are beneficial from an economic and technological perspective. They enable us to increase the rational utilization of wood waste as full-value raw material without the need for their further processing (e.g., fragmentation, gluing, etc.). It should be emphasized that, for many years, there has been a growing interest in the use of wood waste, mainly in the wood, paper, polymer-wood composite, and energy industries. This is due to the necessity of sustainable management of this type of waste, whose resources increase with the development of the wood industry and the demand for wood-based products. This is evidenced, among others, by current technological trends in the production of wood-based materials and numerous studies indicating the possibility of producing full-value products from recycled wood or directly from primary wood processing [27,28,29,30,31,32,33].

The aim of this study was to apply the waste wood particles (WP) from primary wood processing as a filler of open-cell PUR foams. The effects of wood waste’s introduction on the apparent density, cellular morphology, mechanical and thermal properties of PUR foams were examined and discussed.

This paper is a continuation of the previous research conducted by the authors concerning the possibility of using by-products from wood processing in order to manufacture materials with improved properties, which are used, e.g., in construction and in the production of interior design elements [30,31,32].

## 2. Materials and Methods

### 2.1. Materials

A two-component foam system for the production of open-cell polyurethane thermal insulation PUREX-WG 2017 (Polychem System, Poznań, Poland) was used in the research. One of the components was a polyol (A component). The isocyanate component (B component) was polymeric methylenediphenyl-4,4′-diisocyanate consisting of 31.14% free isocyanate groups (NCO).

**Figure 1:**
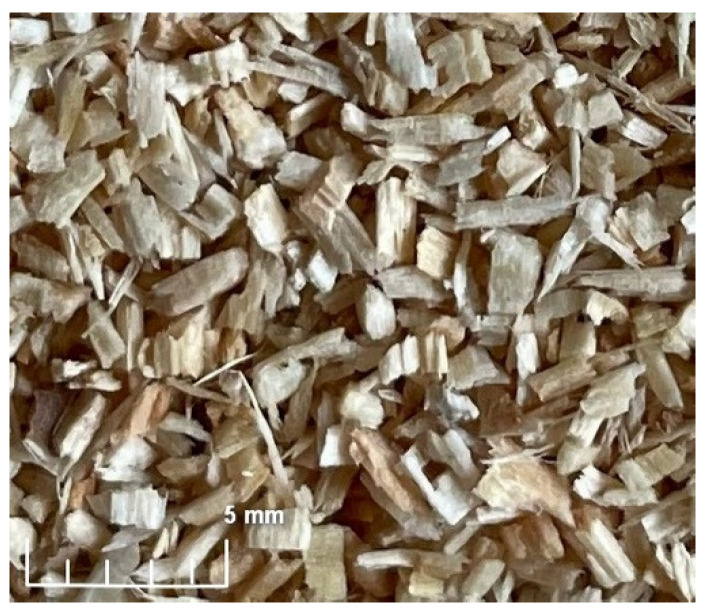
Wood particles used as a filler for PUR foams.

**Figure 2:**
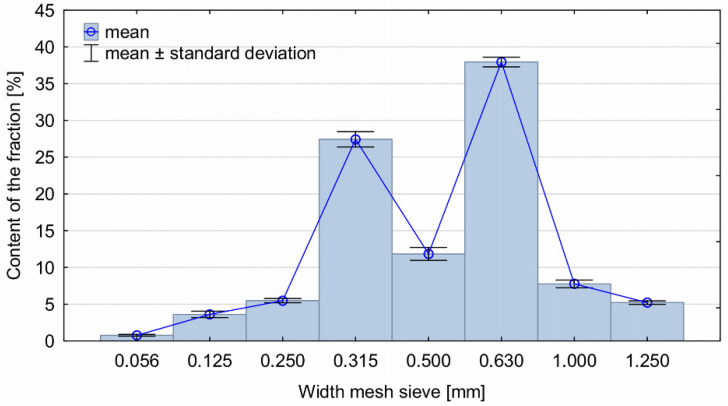
Fractional composition of wood particles added to PUR foams.

Wood particles (WP) representing a dimensional fraction of 0.315–1.25 mm were used as a filler (Figure 1). WP were obtained as a result of sorting sawdust intended for the production of chipboard. The moisture content of WP ranged between 0.2% and 0.5%. Figure 2 presents their fractional composition. The largest shares were observed for the fractions of 0.315 and 0.630 mm. The wood particles were added to the foam at the concentrations of 0, 5, 10, 15 and 20% determined according to a weight ratio. The amount of filler was determined based on preliminary studies and a literature review on the manufacture of biocomposite PUR foams [6,17,34,35].

### 2.2. Synthesis of PUR Composite Foams

**Figure 3:**
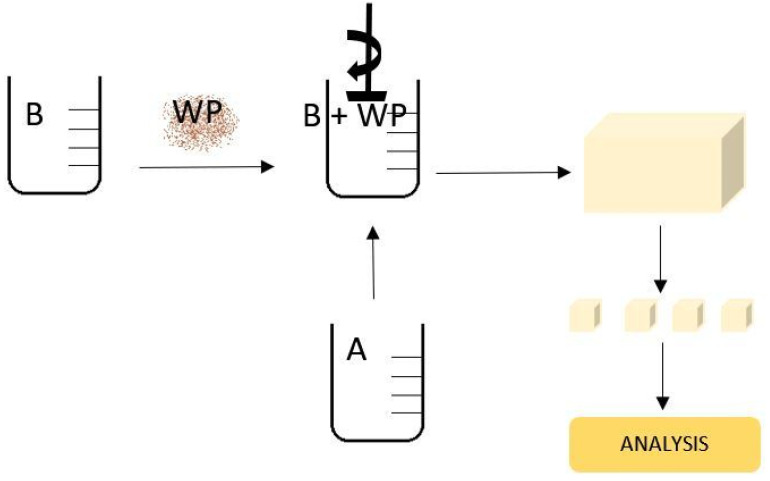
Scheme of PUR manufacturing (A—polyol; B—isocyanate; WP—wood particles).

The course of foaming of the modified PUR foams was characterized on the basis of the times of the successive stages of this process and the maximum foaming temperature. For this purpose, the foam components were mixed in a weight ratio premised in accordance with the manufacturer’s recommendations, i.e., A:B = 100:100. The reaction mixture was prepared by mixing the appropriate amounts of wood particles with component B and then adding component A (Figure 3). The reaction mixture was stirred with a low-speed mechanical stirrer at 1200 rpm for 10 s, at a temperature of 23 °C, and then poured into a form with internal dimensions of 250 × 250 × 130 mm^3^. After that, PUR-WP composites were allowed to grow and were left at room temperature for 24 h. Each foam variant was prepared in two replicates. The obtained foams were cut with a band saw (Holzstar, Hallstadt, Germany) into specimens of dimensions necessary for testing their properties.

### 2.3. Kinetic of PUR Foaming

The influence of wood filler on the foaming process of PUR foams was determined by measuring the following times:

- start of growth*—*time when the volume of the reaction mixture started to increase;
- gelling—the time after which it was possible to remove the so-called “polyurethane thread”;
- growth—the time after which the maximum foam growth was achieved;
- tack-free—the time measured until the foam solidified completely.

The foaming temperatures were measured with a thermocouple immersed in the reaction mixture. The temperature was always read after the foam growth was completed. At the end, the average of 5 individual measurements was evaluated.

### 2.4. Characterization of PUR Sample

The density of neat PUR foam and PUR-WP composites, defined as the ratio of the sample mass to its volume, was determined in accordance with the PN-EN ISO 845 standard. Samples with the dimensions 50 × 50 × 50 mm^3^ were used. The samples were measured with a thickness gauge with an accuracy of 0.01 mm and weighed on an analytical weight with an accuracy of 0.001 g.

The compressive strength in parallel direction to the foam’s growth was investigated in accordance with the recommendations of EN 826 standard using a Tinius Olsen H10KT testing machine (Tinius Olsen Ltd., Salfords, UK). The test covered foam samples with dimensions of 50 × 50 × 50 mm^3^, which were compressed in the direction of foam growth at a rate of 5 mm/min and the load cell of 250 N. The compressive strength (σ_10%_) was defined as the maximum compressive force achieved when the relative deformation at deflection was less than 10% (based on the initial cross-sectional area of the specimen). The means of the compressive strength were evaluated based on the 7 individual measurements.

The thermal conduction coefficient was determined using a heat flux density sensor type ALMEMO 117 company Ahlborn (Holzkirchen, Germany), with plate dimensions of 100 × 30 × 3 mm^3^. The average values of the thermal conduction coefficient were evaluated on the basis of 5 individual measurements. A detailed description of the research method was presented in the previous works by the authors [36,37].

In order to determine the cellular structure, a scanning electron microscope (SEM) and a light microscope were used. SEM analyses were carried out with the use of an SU3500 Hitachi microscope. The images of the sample plane were prepared using a computer image analyzer equipped with a stereoscopic optical microscope (Motic SMZ-168, Hongkong, China) and a camera (Moticam 5.0, Barcelona, Spain). The image of the structure was transferred by a camera to the monitor screen, and then pictures were taken using the Motic Images Plus 3.0 program (Hongkong, China). The cell sizes of pure PUR foam and PUR-WP composite foams were determined using the same equipment. 

The samples with the dimensions 50 × 50 × 50 mm^3^ were collected in order to evaluate the dispersion of WP in PUR foam using computer tomography which is a type of X-ray tomography allowing to obtain cross-sections of the examined object. Scanning was performed with the use of a Hyperion X9Pro tomography, with objects scanned at a resolution of 0.3 mm at a lamp voltage of 90 kV.

The yielded test results of PUR foams with wood particles addition were analyzed statistically using STATISTICA software v.13.1(StatSoft Inc., Tulsa, OK, USA). Mean values of the parameters were compared in a one-factor analysis of variance—post hoc Tukey’s test allowed us to distinguish homogeneous groups of mean values for each parameter for *p* = 0.05.

## 3. Results and discussion

### 3.1. The Impact of WP Filler on PUR Foams Manufacture

**Figure 4:**
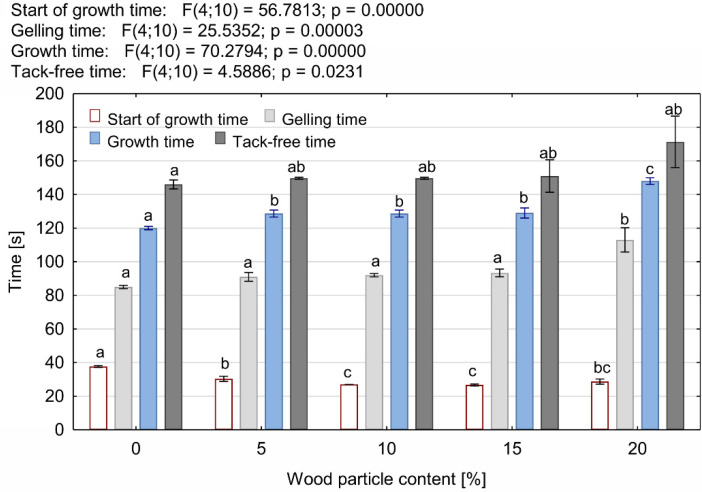
Times characterizing the foaming process of PUR-WP composition depending on wood particle content. Different letters indicate homogeneous groups of mean values determined by one-factor ANOVA with Tukey’s test.

**Figure 5:**
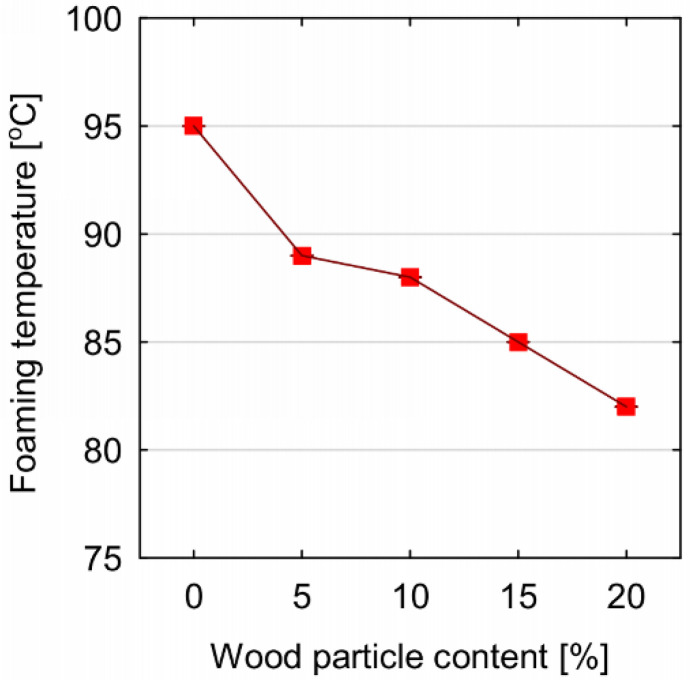
Values of the maximum foaming temperature of PUR-WP composition depending on wood particle content.

The parameters characterizing the foaming process of the tested foams are presented in Figure 4 and Figure 5. It can be concluded that the addition of wood particles to the open-cell PUR foam influences the kinetics of the foaming process of the PUR-WP composition. It was observed that the addition of this type of filler accelerates the onset of foam growth. This phenomenon is particularly evident in the case of PUR-WP compositions containing 10% and 15% of particles. In the case of these variants, a reduction in the starting time by approx. 29% was observed. These statistically significant differences were confirmed by the post hoc test. Statistical analysis allowed for the identification of four different groups of average foam expansion start times for the tested variants. However, mixing the foam components with wood particles had an adverse effect, and it led to extensions in the foams increases the growth time and their gelling times. With the maximum addition of wood particles, the growth time was extended by 23%, and the gel time by 33%. The extension of these times is the effect of slowing down the exothermic reaction, which is also evidenced by the decrease in the maximum foaming temperature of the foams. As shown in Figure 5, the addition of wood particles to the components of foamed PUR foam caused a decrease in the foaming temperature from 95 to 82 °C. According to the literature, the reduction in the maximum foaming temperature of PUR foams results from the reduced amount of heat generated during the reactions occurring in the latent stages and growth, used at the stage of foam stabilization and maturation. In addition, as shown in previous studies, organic fillers introduced into PUR foams can absorb some of the heat generated during synthesis, thus lowering the temperature of their foaming [19,38]. An insufficient amount of heat could slow down the cross-linking reaction and, consequently, extend the time required to achieve a track-free time, which is also confirmed by the data presented in Figure 4 [9]. Moreover, the presence of the wood particles and their relatively large dimensions undoubtedly limited both the growth of the foam cells and the susceptibility of the composition to the foaming process. According to Strąkowska et al. [24], the presence of fillers also limits the mobility of the polymer and the speed of the polymerization reaction. Moreover, as reported by Członka et al. [39], hydroxyl groups present in lignocellulosic fillers can react with highly reactive isocyanate groups. This affects the stoichiometry of the system and reduces the number of isocyanate groups capable of reacting with water, which results in a reduction in the amount of CO_2_ released.

### 3.2. Density, Thermal Conductivity and Microstructure of PUR Foams

**Figure 6:**
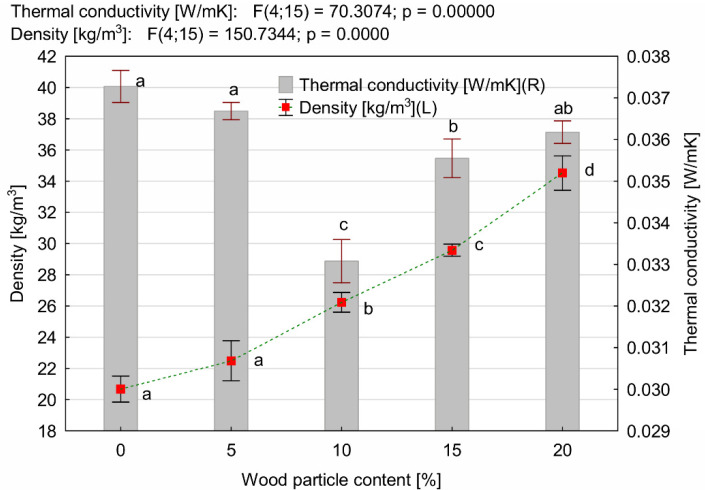
Thermal conductivity and density of PUR-WP composition depending on wood particle content. Different letters indicate homogeneous groups of mean values determined by one-factor ANOVA with Tukey’s test.

As expected, the addition of the wood particles causes a significant increase in the density of the PUR-WP composite. The apparent density of pure PUR foam was 20 kg/m^3^ (Figure 6). The introduction of 5% wood filler particles into its structure caused a slight increase in its density; however, the HSD Tukey test did not confirm statistically significant differences. The mean density values obtained for these two variants belong to the same homogeneous groups. A significant increase in the density of the produced foams was noticed only when larger amounts of wood particles were used, i.e., from 10% and more. With their addition in the amount of 20%, the average apparent density of the foam was 34.6 kg/m^3^, i.e., it increased in comparison to pure foam by as much as 73%.

Along with the increase in the density of the tested foams, the statistically significant changes in their thermal insulation, determined by the thermal conductivity coefficient λ, were also observed. This is confirmed by the results of the post hoc test and the homogeneous groups of mean values λ distinguished on its basis. Moreover, the test probability level was significantly lower than the assumed level of statistical significance, i.e., <0.05. As follows from the data presented in Figure 6, the use of wood particles as a filler for PUR foam at an amount of up to 10% leads to the reduction in the average λ value by approx. 10%, which proves the improvement of thermal insulation of this type of foam.

Unfortunately, a further increase in the wood particle content results in a gradual increase in the value of λ. In the case of the 15% addition of wood filler, the value of λ was lower than the coefficient which the pure foam was characterized by, but higher than that of foams with 10% of wood particles. It should be noted that even with the maximum concentration of wood particles used in the tests (i.e., 20%), the value of the thermal conductivity coefficient was at a level comparable to that of pure PUR foam. Similar observations were made by Tao et al. [34]. The authors noted a decrease in the value of the coefficient λ by as much as 50%, while higher amount of lignocellulosic fibers (up to 20 php) resulted in a gradual increase in thermal conductivity to a level exceeding that of pure PUR foam. 

**Figure 7:**
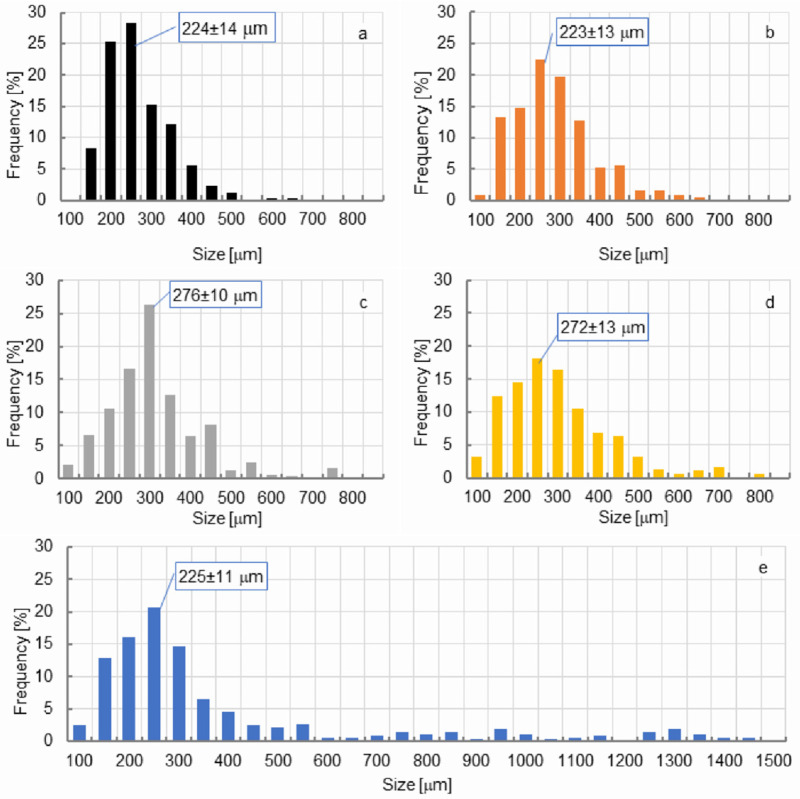
Cell size and cell size distribution of PUR foam depending on wood particle (WP) content: (**a**) control; (**b**) 5%; (**c**) 10%; (**d**) 15%; (**e**) 20%.

**Figure 8:**
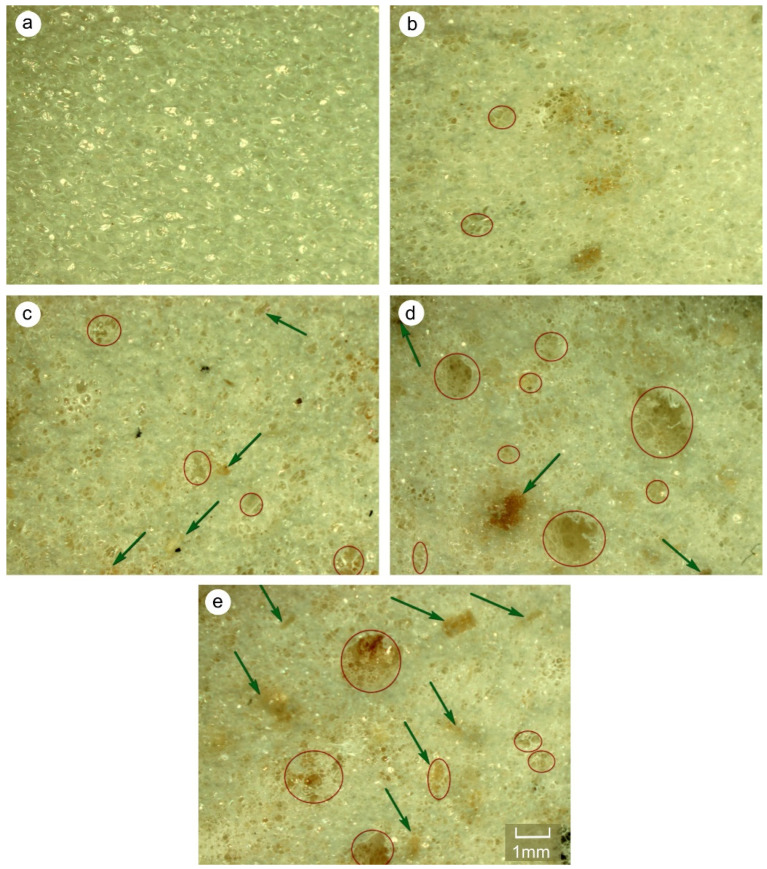
Light microscope photos of PUR samples with addition of different amount of WP: (**a**) control; (**b**) 5%; (**c**) 10%; (**d**) 15%; (**e**) 20% (green arrow—wood particles; red circle—structure disorders, large pores). All scale bars of figures are the same.

**Figure 9:**
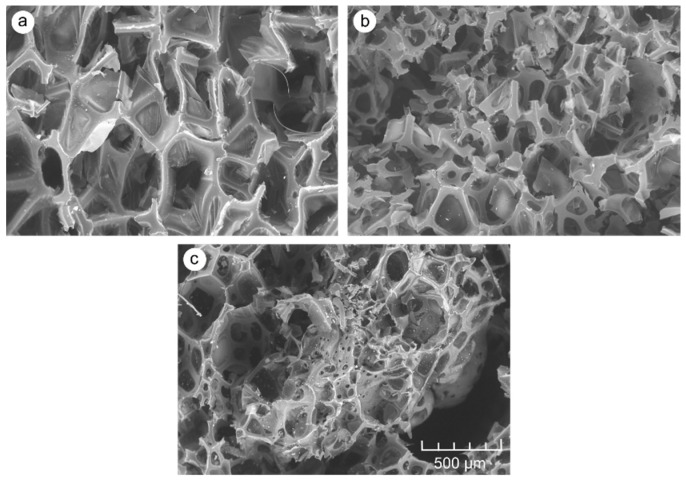
SEM images of PUR samples: (**a**) control sample; (**b**) 10% WP addition; (**c**) 15% WP addition. All scale bars of figures are the same.

This method of shaping the thermal insulation properties of the tested PUR-WP compositions may result mainly from disturbances in the cell structure of the foams. This might be manifested by changes in the distribution of cell sizes. As shown in the literature, such changes have a significant impact on the insulation properties and mechanical strength of PUR foams. As shown in Figure 7, pure PUR foam is characterized by a uniform cell size distribution, mainly within the range of 150–450 µm. The mean cell size in the range of the highest frequency is 224 µm. Introducing relatively small amount of wood particles (i.e., 5 wt.%) to the system reduces the number of cells in the size range of 200–250 µm. At the same time, it increases the number of cells above this range, i.e., within the range of 300–450 µm. However, the mean cell size with the highest frequency is still in the range of 200–250 µm. A further increase in the proportion of wood shavings in the PUR-WP composition in the amount of up to 10% of the weight results in the shifting of the mean cell size from 250 to 300 µm (average cell size 276 µm). However, taking into account the photos taken with a light microscope and SEM (Figure 8 and Figure 9), it can be concluded that despite the noted changes in the cell size distribution, the structure of foams with the 5% and 10% wood filler added is relatively well-developed, which allows for high thermal insulation parameters. The insulating properties of the wood filler itself are likely to be important as well. Wood itself is an excellent insulator, and also has a heat capacity greater than the PUR foam.

A further increase in the amount of wood filler to 15% and 20% causes a significant disturbance of the foam structure and formation of larger and more irregular pores. In these variants, the cell structure of the composite foams is disturbed by the presence of cells sized above 450 µm. This is particularly visible in the case of variants containing 20% of wood particles (Figure 7 and Figure 8), although a collapse of the cellular structure of the foam is visible even with a filler content of 15%. The presence of larger cells and damage to the structure of the foams result in increased air permeability. As a result, it increases the heat transfer and thus reduces the thermal insulation of this type of foam [6,34]. Similar observations in the case of the modification of closed-cell PUR foam with various types of lignocellulosic fillers were made by Strąkowska et al. and Członka et al. [24,39]. Additionally, as shown in the literature, such disturbances in the morphology of the PUR foam due to the introduction of the organic filler, such as straw particles, may result from poor interfacial adhesion between the polymer matrix and the filler surface, which consequently disrupts the foaming process and, as a result, the structure of modified PUR foams [40]. Additionally, according to Sung et al. [41], during the cell structure formation of PUR foams, the interaction between the filler surface and the polymer matrix can determine the final average cell sizes. The higher the hydrophilicity of the filler surface, as in the case of wood filler, the larger the cell sizes in the microstructure of foams. Moreover, the filler particles may constitute a nucleating agent and cause the nucleation pattern to change from homogeneous to heterogeneous and reduce the nucleation energy. For this reason, smaller cells are formed in the foam structure [42,43]. In the case of our research, the formation of cells of a small size, i.e., about 100 and 150 µm in size, was also noted. 

This can also be confirmed by the analysis of the cell size distribution and SEM (Figure 7 and Figure 9). It also proves a significant differentiation of the cell size distribution of PUR-WP composite foams. The formation of this type of cell may result from the attachment of filler particles to the foam cell, which leads to damage and weakening of the foam microstructure, and thus lowers its strength. 

**Figure 10:**
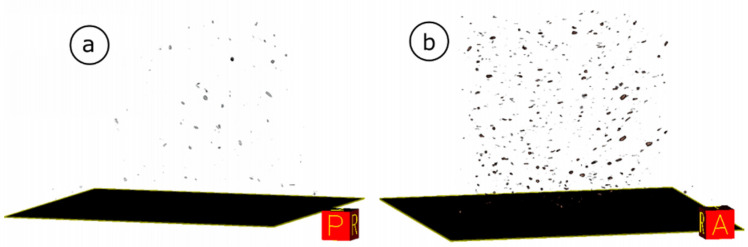
Image of foam with the addition of wood particles made with a computer tomograph: (**a**) 5% WP addition; (**b**) 20 addition WP.

Analyzing the structure of the produced foams, attention should also be paid to the dispersions of wood particles. As a rule, fillers with smaller particle sizes (e.g., nano-scale) tend to agglomerate, which also interferes with the foaming process and the morphology of PUR foams. The filler of relatively large dimensions and irregular shape used in the research allows for a high degree of dispersion of its particles in the polyurethane matrix. This is evidenced by the 3D photos of foams with 5% and 20% addition of wood particles. The photos were made with the use of the computed tomography technique, presented in Figure 10.

### 3.3. Compressive Strength

**Figure 11:**
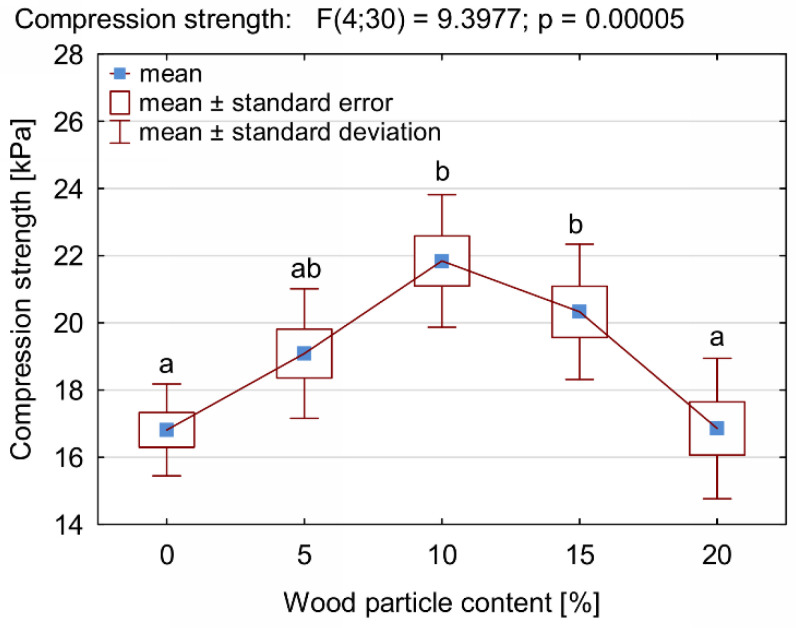
Compression strength of PUR-WP composition depending on wood particle content. Different letters indicate homogeneous groups of mean values determined by one-factor ANOVA with Tukey’s test.

**Figure 12:**
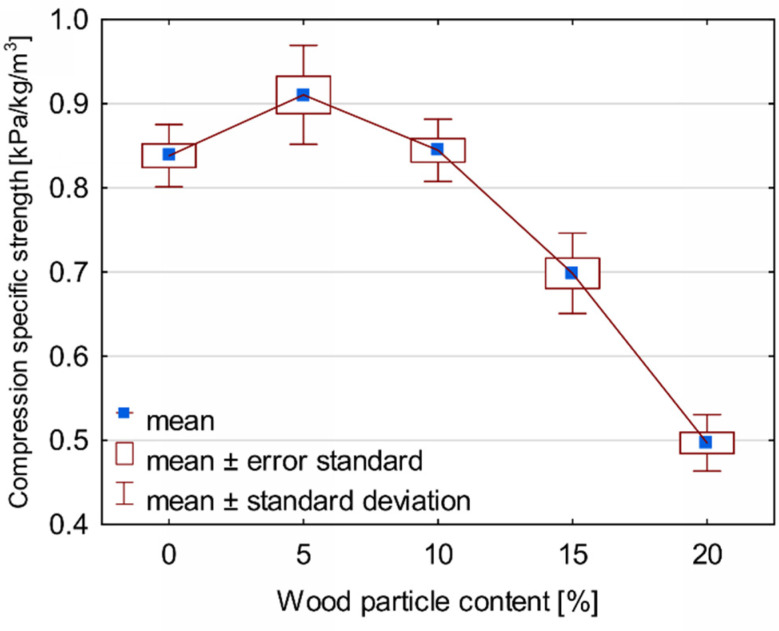
Compression specific strength of PUR-WP composition depending on wood particle content.

The results of the structure and thermal insulation properties of the produced foams correspond with the results of the analysis of the compressive strength measurements. As shown in Figure 11 and Figure 12, the maximum increase in the mean value of compressive strength was recorded for the composition with a 10% share of foam wood particles—an increase in σ_10%_ by approx. 30% in comparison with control PUR foam. With a 15% addition of wood filler, a tendency towards a reduction in compressive strength can be noticed, but it should be emphasized that the HSD Tukey analysis did not confirm the statistically significant differences noted for the compositions containing 10% and 15% of wood particles (the same homogeneous group b). A statistically significant decrease in compressive strength was noticed only for the composition containing 20% wood particles. It should be emphasized that in this case, the compressive strength was comparable to control samples (the same homogeneous group-a). The increase in the compressive strength of compositions containing up to 10% wood filler can be explained by an increase in the apparent density of the tested foams. At the same time, the foam still has a well-developed cell structure, which is also confirmed by the analysis of the cell size distribution of the tested foams and the photos in Figure 7, Figure 8 and Figure 9.

Despite a significant increase in the apparent density of foams, a further increase in the amount of wood particles reduced the strength of PUR-WP. Such shaping of the compressive strength of the tested compositions proves that this type of parameter is influenced not only by the apparent density of the foam, but also by its structure. This is confirmed by the research of other authors [38,39,44] and the additionally estimated specific compressive strength of the produced PUR-WP compositions, which is defined as the ratio of the compressive strength σ_10%_ to the density of the tested foams [39]. As demonstrated by the analysis, the use of waste wood particles of such large dimensions as PUR foam filler increases the specific compressive strength by up to 5%. Above this amount, the value of this parameter gradually decreases. 

This is due to the fact that foams with 5% of wood filler have a homogeneous structure with a narrow range of cell size distribution (like pure foam). The proper filler dispersion with a well-formed foam structure probably facilitates the transfer of strain under compressive load and thus increases its strength. As mentioned earlier, higher filler additions result in a broadening of the range of the cell size distribution and the presence of numerous structural disorders (especially at 15% and 20% of the WP activity). Despite the increase in density, these foams are characterized by lower compressive strength.

## 4. Conclusions

This article presents the influence of wood particles as a filler in PUR foams. The WP was added in different amounts, i.e., 0, 5, 10, 15 and 20% to PUR and apparent density, cellular morphology, mechanical properties and thermal property were conducted. As expected, the addition of the wood particles causes a significant increase in the density of the PUR-WP composite. A significant increase in the density of the produced foams was noticed only when larger amounts of wood particles were used, i.e., from 10% and more. Along with the increase in the density of the tested foams, statistically significant changes in their thermal insulation were observed. The addition of wood filler in the amount of 10% allows us to improve the insulation properties of PUR foam, which is manifested by a decrease in the value of the thermal conductivity coefficient by 10%. It should be noted that even with the maximum amount of wood particles used in the tests (i.e., 20%), the value of the thermal conductivity coefficient was at a level comparable to that of pure PUR foam. Moreover, the results of the compressive strength in the parallel direction to the foam’s growth showed that addition of 10% WP to the foam lead to the increase in σ_10%_ by approx. 30% in comparison with the control PUR foam. The increase in the compressive strength of compositions containing up to 10% can be explained by an increase in the apparent density of the tested foams. Thus, the conducted studies indicated the possibility of using wood waste as fillers for PUR foams with open-cell structure. Such composite foams with 10 wt.% of waste wood particles from primary wood processing can be used as thermal insulation of open diffusivity building partitions in modern prefabricated buildings.

## Data Availability

The data presented in this study are available on request from the corresponding author.
